# Optimization of Condition Monitoring Decision Making by the Criterion of Minimum Entropy

**DOI:** 10.3390/e21121193

**Published:** 2019-12-04

**Authors:** Ahmed Raza, Vladimir Ulansky

**Affiliations:** 1 Projects and Maintenance Section, The Private Department of the President of the United Arab Emirates, Abu Dhabi 000372, UAE; ahmed.awan@dopa.ae or; 2 Research and Development Department, Mathematical Modelling & Research Holding Limited, London W1W 7LT, UK; 3 Department of Electronics, Robotics, Monitoring Technology, and IoT, National Aviation University, 03058 Kyiv, Ukraine

**Keywords:** condition monitoring, false-positive, false-negative, Shannon entropy, preventive maintenance threshold, minimum entropy

## Abstract

Condition-based maintenance (CBM) is a promising technique for a wide variety of deteriorating systems. Condition-based maintenance’s effectiveness largely depends on the quality of condition monitoring. The majority of CBM mathematical models consider perfect inspections, in which the system condition is assumed to be determined error-free. This article presents a mathematical model of CBM with imperfect condition monitoring conducted at discrete times. Mathematical expressions were derived for evaluating the probabilities of correct and incorrect decisions when monitoring the system condition at a scheduled time. Further, these probabilities were incorporated into the equation of the Shannon entropy. The problem of determining the optimal preventive maintenance threshold at each inspection time by the criterion of the minimum of Shannon entropy was formulated. For the first time, the article showed that Shannon’s entropy is a convex function of the preventive maintenance threshold for each moment of condition monitoring. It was also shown that the probabilities of correct and incorrect decisions depend on the time and parameters of the degradation model. Numerical calculations show that the proposed approach to determining the optimal preventive maintenance threshold can significantly reduce uncertainty when deciding on the condition of the monitoring object.

## 1. Introduction

The concept of “entropy” is widely used in various fields of science. Its discoverer, Clausius, introduced this concept in the early 1850s for highly specific thermodynamic purposes. He proved a theorem that states that the amount of heat received by the system in any circular process, divided by the absolute temperature at which it was received, is not positive.

Boltzmann, between 1872 and 1875, introduced the concept of the entropy of a thermodynamic system that is defined as the product of Boltzmann’s constant and natural logarithm of the number of different microscopic states corresponding to a given macroscopic state.

Shannon, in 1948, proposed using the concept of entropy in information theory [1]. The Shannon formula calculates information binary entropy for independent random events with *m* possible states distributed with probabilities $\vec{p}=\bar{p_{1},\;p_{m}}$:

(1) $$H\left(\vec{p}\right)=-\sum_{j=1}^{m}p_{j}\log_{2}p_{j}$$

The Shannon entropy equivalently measures the amount of uncertainty represented by a probability distribution $\vec{p}=\bar{p_{1},\;p_{m}}$. Initially, only communication theory used the concept of Shannon entropy. However, subsequently, the Shannon entropy began to be used in many different fields of science and technology such as machine learning [2], biomedical informatics [3], reliability [4], prognostics [5], fault detection [6], condition monitoring [7], maintenance [8], fingerprint recognition [9], geosciences [10], fatigue damage modeling [11], and many others.

Such widespread use of the Shannon entropy concept indicates its great potential in solving various problems of science and technology. As mentioned above, Shannon entropy has also been used as a metric in the problems of optimizing condition monitoring and maintenance [7,8]. In condition monitoring tasks, entropy usually represents a metric for the selection of informative data received from sensors [12,13]. In condition-based maintenance (CBM) tasks, the Shannon entropy is used to evaluate the degradation process [14,15]. However, such use of Shannon entropy in CBM as a metric seems wholly insufficient. Indeed, the main objectives of the CBM include (1) the accumulation of statistical data for each component of equipment, (2) determination of the equipment component degradation rate, (3) selection of the most effective inspection schedule, and (4) minimization of the failure risk for the selected inspection schedule [16]. As can be seen, the concept of entropy in published studies is still being used to solve tasks associated with the first and second intermediate objectives. But, the Shannon entropy has not yet been used to solve the more significant third and fourth CBM objectives. There is an explanation for this fact. Two preliminary tasks should be solved to use Shannon entropy for reaching the third and fourth objectives. Firstly, it is necessary to choose or derive indicators of the maintenance’s effectiveness that would include the probabilities of correct and incorrect decisions made by the results of condition monitoring; and, secondly, these probabilities should be functions of operational time and parameters of the degradation model. However, as the related literature shows (see Section 2), all relevant studies assume that either the condition monitoring is perfect or the probabilities of correct and incorrect decisions are constant and do not depend on the parameters of the degradation model. Under these assumptions, when using CBM, it is impossible to solve correctly the problems of determining the optimal inspection schedule and minimizing the probability of failure in the upcoming time interval. The latter is because assuming perfect monitoring or assuming the error probabilities to be constant is impossible to choose the optimal threshold for preventive maintenance, according to which potentially unreliable systems would be rejected.

The purpose of this study is to present a CBM decision-making method based on determining the optimal preventive maintenance threshold on the criterion of minimal Shannon entropy for each inspection time. A mathematical model of CBM was developed which considers the probabilities of correct and incorrect decisions made when checking system operability over the next interval of operation at scheduled inspection times. Contrary to previous studies, these probabilities depend on operational time, parameters of the degradation model, and the preventive maintenance threshold. Then, these probabilities were incorporated into the formula of Shannon entropy. As a result, Shannon entropy depended on the scheduled time of condition monitoring and threshold of preventive maintenance. Further, the problem was formulated to determine the optimal threshold by the criterion of minimum Shannon entropy for each of the inspection times. The proposed approach significantly reduces the probability of system failure in the interval between inspections due to the rejection of potentially unreliable systems. Finally, numerical calculations for a degrading radar power supply are presented to illustrate the application and advantages of the proposed method.

The organization of the article is as follows: Section 2 provides a literature review on the modeling of CBM. In Section 3, the quantification of uncertainty when monitoring a system’s condition is conducted. Section 4 considers the Shannon entropy of imperfect condition monitoring. The optimality criterion of preventive maintenance thresholds is presented in Section 5. Section 6 examines a model of the stochastic degradation process. Section 7 presents the results and discussion. In Section 8, the conclusions are formulated. Abbreviations, nomenclature, and references are given at the end of the article.

## 2. Literature Review

Maintenance based on condition monitoring is currently considered as a promising approach for improving operational reliability and reducing the operating costs of various deteriorating systems. A growing interest in CBM is manifested by a large number of studies devoted to various mathematical models and methods of optimization. The majority of the existing CBM models with scheduled inspections can be conditionally divided into two groups: CBM models with perfect inspections and CBM models with imperfect inspections.

First, let us consider CBM models with perfect inspections. Chen et al. [17] considered an optimal replacement strategy for CBM with optimal inspection intervals for the case when degradation corresponds to an inverse Gaussian process with random effects. Abdel-Hameed [18,19] presented a model of optimal periodic inspections based on the class of increasing Markov processes. The inspection periodicity and preventive maintenance threshold are considered variables. Grall et al. [20] considered a system that is subjected to stochastic degradation and monitored using inspections. Corrective or preventive maintenance is carried out when the deteriorating process reaches either the failure threshold or preventive maintenance threshold. Dieulle et al. [21] proposed a mathematical model to investigate the joint influence of the preventive maintenance threshold and inspection schedule on the total costs of the system maintenance. Deloux et al. [22] considered an approach to the construction and optimization of CBM policy for an accumulative degradation system. The optimization target function is the total cost of various inspections, replacements, and idle time. Grall et al. [23] considered a CBM structure for a gradually degrading single-unit system. The proposed decision-making model is used to determine the optimal inspection schedule and, if necessary, the replacement times as well. Huynh et al. [24] considered CBM of a single-unit system subject to dependent failures due to the fact of deterioration and traumatic shock events. Wang et al. [25] introduced the maintenance scheduling threshold for organizing the maintenance resources according to the system state. The maintenance scheduling threshold is used as a controlled variable in combination with the preventive maintenance threshold and failure threshold. Guo et al. [26] considered a CBM strategy with three possible actions: periodic inspection, preventive maintenance, and corrective maintenance. Liu et al. [27] considered a maintenance policy for degrading systems with state-dependent operating costs. The system is replaced when the level of its degradation exceeds the preventive maintenance threshold. Flage et al. [28] considered a model determining an optimal inspection and maintenance scheme for a one-unit system with a stochastic degradation process. Deloux et al. [29] proposed modeling the influence of a random operating environment on the behavior of a system with randomization of gamma-process degradation parameters.

In the analyzed CBM models [17,18,19,20,21,22,23,24,25,26,27,28,29], the authors assumed perfect inspections as a result of which the condition of the system is determined error-free. However, in reality, the inspections are imperfect, and incorrect decisions about system condition are possible.

He et al. [30] examined a maintenance model with periodic imperfect inspections. When inspecting the system, its failure is detected with the probability *p* ∈ (0, 1). After failure detection, a corrective maintenance of the system is performed. If no failure was detected over a specified time interval, the system is replaced with a new one. Kallen and Noortwijk [31] considered a decision-making model for the case of periodic inspections of the system condition which minimizes the expected average cost per year. The observed stochastic process includes the original process and a normally distributed measurement error. Newby and Dagg [32] considered a CBM model in which the measurement result includes the initial process of system degradation along with a normally distributed measurement error. Ye et al. [33] considered a CBM model which utilizes a stochastic Wiener process to model degradation with measurement error. Within this model, the distribution of the remaining useful life (RUL) is calculated which is used to make decisions about restoring or using the system. Tang et al. [34] also proposed a novel RUL prediction method for lithium-ion batteries based on the Wiener process with measurement error which can be used for optimizing CBM. Lam [35] considered a CBM model of a deteriorating system with non-perfect inspections. That is, an inspection is associated with the probability of detection and probability of false alarm. Badıa et al. [36] proposed a maintenance model where the result of inspection may give a wrong result.

Maintenance models with imperfect inspections proposed in References [37,38,39] considered two types of errors: false-positives with conditional probability α and false-negatives with conditional probability β and, accordingly, true-positives and true-negatives with conditional probabilities 1 − α and 1 − β. These studies did not consider any preventive maintenance threshold when checking the system, and the conditional probabilities of incorrect decisions α and β were constant. They did not depend on the parameters of the system degradation process. However, in reality, the error probabilities when checking the deteriorating system condition are not constant coefficients but depend on the time and parameters of the degradation process [40].

The conducted analysis of the CBM mathematical models shows that a large number of research articles are devoted to solving various problems associated with condition monitoring and decision-making. The published studies pay particular attention to the determination of the optimal preventive maintenance threshold, optimal inspection schedule, the trustworthiness of inspections, optimization criteria as well as degradation process models. The majority of published CBM mathematical models consider perfect inspections, in which the system condition is determined error-free. The mathematical models of maintenance with imperfect inspections are based on the decision rule, aimed at rejecting only systems that are inoperable at the time of condition monitoring. The drawback of this decision rule is the impossibility of rejecting the systems that may fail within the operation interval before the next time point of condition monitoring. Also, some mathematical models assume that the probabilities of incorrect decisions when monitoring the system condition are constants and do not depend on the time and degradation process parameters which does not reflect the real conditions.

## 3. Quantification of Condition Monitoring Uncertainty at Successive Times

Let us assume that the condition of the system is determined by the value of a state parameter *Y*(*t*), which is a non-stationary continuous-time stochastic process with monotonically increasing realizations. The system operates in an infinite time interval and is monitored at successive times $t_{1},t_{2},\ldots ,t_{i},\;\ldots$ (*i* = 1, 2, …), where $t_{0}=0$. The measurement result of the state parameter *Y*(*t*) at time $t_{i}$ is expressed as

(2) $$\Xi \left(t_{i}\right)=Y\left(t_{i}\right)+N\left(t_{i}\right)$$

where $N(t_{i})$ is the random noise or measurement error at time $t_{i}$.

Further, we assume that random variables $Y(t_{i})$ and $N(t_{i})$ are independent.

When checking the system condition at time $t_{i}$, we introduce the following decision rule. If $\xi (t_{i})<PT_{i}$, the system is judged as operable in the interval $\left(t_{i},t_{i+1}\right)$, where $\xi (t_{i})$ is the realization of $\Xi (t_{i})$ at time $t_{i}$, $PT_{i}$ is the preventive maintenance threshold $\left(PT_{i}\leq FT\right)$ at time $t_{i}$, and FT is the functional failure threshold. If $\xi (t_{i})\geq PT_{i}$, the system is judged as inoperable in the interval $\left(t_{i},t_{i+1}\right)$. Therefore, the decision rule is intended to reject any system that is not operable for use in the next interval of operation.

From the perspective of the applicability of the system that should operate in the interval $\left(t_{i},t_{i+1}\right)$, when monitoring *Y*(*t*) at time $t=t_{i}$, one of the following mutually exclusive events may occur:

(3) $$\Gamma_{1}\left(t_{i},t_{i+1}\right)=\left\{Y\left(t_{i+1}\right)<FT\cap \Xi \left(t_{i}\right)<PT_{i}\right\}$$

(4) $$\Gamma_{2}\left(t_{i},t_{i+1}\right)=\left\{Y\left(t_{i+1}\right)<FT\cap \Xi \left(t_{i}\right)\geq PT_{i}\right\}$$

(5) $$\Gamma_{3}\left(t_{i},t_{i+1}\right)=\left\{Y\left(t_{i}\right)<FT\cap Y\left(t_{i+1}\right)\geq FT\cap \Xi \left(t_{i}\right)<PT_{i}\right\}$$

(6) $$\Gamma_{4}\left(t_{i},t_{i+1}\right)=\left\{Y\left(t_{i}\right)<FT\cap Y\left(t_{i+1}\right)\geq FT\cap \Xi \left(t_{i}\right)\geq PT_{i}\right\}$$

(7) $$\Gamma_{5}\left(t_{i},t_{i+1}\right)=\left\{Y\left(t_{i}\right)\geq FT\cap \Xi \left(t_{i}\right)<PT_{i}\right\}$$

(8) $$\Gamma_{6}\left(t_{i},t_{i+1}\right)=\left\{Y\left(t_{i}\right)\geq FT\cap \Xi \left(t_{i}\right)\geq PT_{i}\right\}$$

where $\Gamma_{1}\left(t_{i},t_{i+1}\right)$ is the joint occurrence of the following events: the system is operable over the time interval $\left(t_{i},t_{i+1}\right)$ and is judged as operable over the time interval $\left(t_{i},t_{i+1}\right)$ at inspection time $t_{i}$; $\Gamma_{2}\left(t_{i},t_{i+1}\right)$ is the joint occurrence of the following events: the system is operable over the time interval $\left(t_{i},t_{i+1}\right)$ and is judged as inoperable over the time interval $\left(t_{i},t_{i+1}\right)$ at inspection time $t_{i}$; $\Gamma_{3}\left(t_{i},t_{i+1}\right)$ is the joint occurrence of the following events: the system is operable at inspection time $t_{i}$, fails within the interval $\left(t_{i},t_{i+1}\right)$, and is judged as operable over the interval $\left(t_{i},t_{i+1}\right)$ at inspection time $t_{i}$; $\Gamma_{4}\left(t_{i},t_{i+1}\right)$ is the joint occurrence of the following events: the system is operable at inspection time $t_{i}$, fails within the interval $\left(t_{i},t_{i+1}\right)$, and is judged as inoperable over the interval $\left(t_{i},t_{i+1}\right)$ at inspection time $t_{i}$; $\Gamma_{5}\left(t_{i},t_{i+1}\right)$ is the joint occurrence of the following events: the system has failed until inspection time $t_{i}$ and is judged as operable over the time interval $\left(t_{i},t_{i+1}\right)$ at inspection time $t_{i}$; $\Gamma_{6}\left(t_{i},t_{i+1}\right)$ is the joint occurrence of the following events: the system has failed until inspection time $t_{i}$ and is judged as inoperable over the time interval $\left(t_{i},t_{i+1}\right)$ at inspection time $t_{i}$.

Further, the event $\Gamma_{2}\left(t_{i},t_{i+1}\right)$ is called a “false-positive”, and events $\Gamma_{3}\left(t_{i},t_{i+1}\right)$ and $\Gamma_{5}\left(t_{i},t_{i+1}\right)$ are called “false-negative 1” and “false-negative 2”, respectively. The events $\Gamma_{1}\left(t_{i},t_{i+1}\right)$, $\Gamma_{4}\left(t_{i},t_{i+1}\right)$, and $\Gamma_{6}\left(t_{i},t_{i+1}\right)$ correspond to the correct decisions named as “true-positive”, “true-negative 1”, and “true-negative 2”, respectively.

Let us determine the probabilities of the events $\Gamma_{k}\left(t_{i},t_{i+1}\right)$, $k=\bar{1,6}$. By the multiplication theorem on the probability for the event $\Gamma_{1}\left(t_{i},t_{i+1}\right)$ we have:

(9) $$P\left\{\Gamma_{1}\left(t_{i},t_{i+1}\right)\right\}=P\left\{Y\left(t_{i+1}\right)<FT\right\}P\left\{\Xi \left(t_{i}\right)<PT|Y\left(t_{i+1}\right)<FT\right\}$$

where $P\left\{Y\left(t_{i+1}\right)<FT\right\}$ is the a priori probability that the system is in the operable state at time $t_{i+1}$ and $P\left\{\Xi \left(t_{i}\right)<PT_{i}|Y\left(t_{i+1}\right)<FT\right\}$ is the conditional probability of judging the system operable over the interval $\left(t_{i},t_{i+1}\right)$ at the inspection time $t_{i}$ under the condition that the system will not fail up to the time $t_{i+1}$.

For the monotonic stochastic process of degradation, the probability that the system will not fail before time $t_{i+1}$ is the same as the reliability function and is given by:

(10) $$P\left\{Y\left(t_{i+1}\right)<FT\right\}=\int_{-\infty }^{FT}\omega \left(y_{i+1}\right)dy_{i+1}$$

where $\omega (y_{i+1})$ is the probability density function (PDF) of the system state parameter *Y*(*t*) at time $t=t_{i+1}$.

We determine the conditional probability $P\left\{\Xi \left(t_{i}\right)<PT_{i}|Y\left(t_{i+1}\right)<FT\right\}$ by integrating the conditional PDF $\theta \left\{\xi_{i}|Y\left(t_{i+1}\right)<FT\right\}$ of the random variable $\Xi \left(t_{i}\right)$ as follows:

(11) $$P\left\{\Xi \left(t_{i}\right)<PT_{i}|Y\left(t_{i+1}\right)<FT\right\}=\int_{-\infty }^{PT_{i}}\theta \left\{\xi_{i}|Y\left(t_{i+1}\right)<FT\right\}d\xi_{i}$$

Under the assumption that *Y*(*t*) and *N*(*t*) are independent random variables, the conditional PDF $\theta \left\{\xi_{i}|Y\left(t_{i+1}\right)<FT\right\}$ is the convolution of functions $f\left\{y_{i}|Y\left(t_{i+1}\right)<FT\right\}$ and $\varphi (n_{i})$, where $f\left\{y_{i}|Y\left(t_{i+1}\right)<FT\right\}$ is the conditional PDF of random variable *Y*(*t*) at time $t=t_{i}$ under the condition that $Y\left(t_{i+1}\right)<FT$ and $\varphi (n_{i})$ is the PDF of random variable *N*(*t*) at time $t=t_{i}$.

Applying the formula of convolution integral we get:

(12) $$\theta \left\{\xi_{i}|Y\left(t_{i+1}\right)<FT\right\}=\int_{-\infty }^{FT}f\left(y_{i}|Y\left(t_{i+1}\right)<FT\right)\varphi \left(\xi_{i}-y_{i}\right)dy_{i}$$

By substitution of Equation (12) to (11) we obtain:

(13) $$P\left\{\Xi \left(t_{i}\right)<PT_{i}|Y\left(t_{i+1}\right)<FT\right\}=\int_{-\infty }^{FT}f\left(y_{i}|Y\left(t_{i+1}\right)<FT\right)\int_{-\infty }^{PT_{i}}\varphi \left(\xi_{i}-y_{i}\right)d\xi_{i}dy_{i}$$

Making the change of variables $n_{i}=\xi_{i}-y_{i}$ in Equation (13) gives:

(14) $$P\left\{\Xi \left(t_{i}\right)<PT_{i}|Y\left(t_{i+1}\right)<FT\right\}=\int_{-\infty }^{FT}f\left(y_{i}|Y\left(t_{i+1}\right)<FT\right)\int_{-\infty }^{PT_{i}-y_{i}}\varphi \left(n_{i}\right)dn_{i}dy_{i}$$

By the Bayes formula for continuous random variables, we determine the conditional PDF:

(15) $$f\left(y_{i}|Y\left(t_{i+1}\right)<FT\right)=\int_{-\infty }^{FT}\omega \left(y_{i},y_{i+1}\right)dy_{i+1}/\int_{-\infty }^{FT}\omega \left(y_{i+1}\right)dy_{i+1}$$

where $\omega (y_{i},y_{i+1})$ is the joint PDF of random variables $Y(t_{i})$ and $Y(t_{i+1})$.

By substitution of Equation (15) into (14) we get:

(16) $$P\left\{\Xi \left(t_{i}\right)<PT_{i}|Y\left(t_{i+1}\right)<FT\right\}=\frac{\int_{-\infty }^{FT}\int_{-\infty }^{FT}\omega \left(y_{i},y_{i+1}\right)\int_{-\infty }^{PT_{i}-y_{i}}\varphi \left(n_{i}\right)dn_{i}dy_{i}dy_{i+1}}{\int_{-\infty }^{FT}\omega \left(y_{i+1}\right)dy_{i+1}}$$

The final expression for the probability of a true-positive, we obtain by substitution of Equations (16) and (10) into (9).

(17) $$P\left\{\Gamma_{1}\left(t_{i},t_{i+1}\right)\right\}=\int_{-\infty }^{FT}\int_{-\infty }^{FT}\omega \left(y_{i},y_{i+1}\right)\int_{-\infty }^{PT_{i}-y_{i}}\varphi \left(n_{i}\right)dn_{i}dy_{i}dy_{i+1}$$

Applying the multiplication theorem on the probability to the false-positive event (4) gives:

(18) $$P\left\{\Gamma_{2}\left(t_{i},t_{i+1}\right)\right\}=P\left\{Y\left(t_{i+1}\right)<FT\right\}P\left\{\Xi \left(t_{i}\right)\geq PT|Y\left(t_{i+1}\right)<FT\right\}$$

where $P\left\{\Xi \left(t_{i}\right)\geq PT|Y\left(t_{i+1}\right)<FT\right\}$ is the conditional probability of judging the system inoperable over the interval $\left(t_{i},t_{i+1}\right)$ at the inspection time $t_{i}$ under the condition that the system will not fail up to the time $t_{i+1}$.

Integrating the conditional PDF $\theta \left\{\xi_{i}|Y\left(t_{i+1}\right)<FT\right\}$ of a random variable $\Xi \left(t_{i}\right)$ over the range of exceeding the preventive threshold $PT_{i}$, we determine the conditional probability of judging the system inoperable as follows:

(19) $$P\left\{\Xi \left(t_{i}\right)\geq PT_{i}|Y\left(t_{i+1}\right)<FT\right\}=\int_{PT_{i}}^{\infty }\theta \left\{\xi_{i}|Y\left(t_{i+1}\right)<FT\right\}d\xi_{i}$$

By substitution of Equation (12) to (19) we obtain:

(20) $$P\left\{\Xi \left(t_{i}\right)\geq PT_{i}|Y\left(t_{i+1}\right)<FT\right\}=\int_{-\infty }^{FT}f\left(y_{i}|Y\left(t_{i+1}\right)<FT\right)\int_{PT_{i}}^{\infty }\varphi \left(\xi_{i}-y_{i}\right)d\xi_{i}dy_{i}$$

Changing the variables $n_{i}=\xi_{i}-y_{i}$ in Equation (20) results in:

(21) $$P\left\{\Xi \left(t_{i}\right)\geq PT_{i}|Y\left(t_{i+1}\right)<FT\right\}=\int_{-\infty }^{FT}f\left(y_{i}|Y\left(t_{i+1}\right)<FT\right)\int_{PT_{i}-y_{i}}^{\infty }\varphi \left(n_{i}\right)dn_{i}dy_{i}$$

Substituting Equation (15) into (21) gives:

(22) $$P\left\{\Xi \left(t_{i}\right)\geq PT_{i}|Y\left(t_{i+1}\right)<FT\right\}=\frac{\int_{-\infty }^{FT}\int_{-\infty }^{FT}\omega \left(y_{i},y_{i+1}\right)\int_{PT_{i}-y_{i}}^{\infty }\varphi \left(n_{i}\right)dn_{i}dy_{i}dy_{i+1}}{\int_{-\infty }^{FT}\omega \left(y_{i+1}\right)dy_{i+1}}$$

By substituting Equations (10) and (22) into (18), we obtain the following equation for the probability of a false-positive:

(23) $$P\left\{\Gamma_{2}\left(t_{i},t_{i+1}\right)\right\}=\int_{-\infty }^{FT}\int_{-\infty }^{FT}\omega \left(y_{i},y_{i+1}\right)\int_{PT_{i}-y_{i}}^{\infty }\varphi \left(n_{i}\right)dn_{i}dy_{i}dy_{i+1}$$

The probabilities of the events (5)–(8) are derived analogically to the probabilities $P\left\{\Gamma_{1}\left(t_{i},t_{i+1}\right)\right\}$ and $P\left\{\Gamma_{2}\left(t_{i},t_{i+1}\right)\right\}$. Applying some mathematical manipulations to the events (5)–(8), we get:

(24) $$P\left\{\Gamma_{3}\left(t_{i},t_{i+1}\right)\right\}=\int_{FT}^{\infty }\int_{-\infty }^{FT}\omega \left(y_{i},y_{i+1}\right)\int_{-\infty }^{PT_{i}-y_{i}}\varphi \left(n_{i}\right)dn_{i}dy_{i}dy_{i+1}$$

(25) $$P\left\{\Gamma_{4}\left(t_{i},t_{i+1}\right)\right\}=\int_{FT}^{\infty }\int_{-\infty }^{FT}\omega \left(y_{i},y_{i+1}\right)\int_{PT_{i}-y_{i}}^{\infty }\varphi \left(n_{i}\right)dn_{i}dy_{i}dy_{i+1}$$

(26) $$P\left\{\Gamma_{5}\left(t_{i},t_{i+1}\right)\right\}=\int_{FT}^{\infty }\omega \left(y_{i}\right)\int_{-\infty }^{PT_{i}-y_{i}}\varphi \left(n_{i}\right)dn_{i}dy_{i}dy_{i+1}$$

(27) $$P\left\{\Gamma_{6}\left(t_{i},t_{i+1}\right)\right\}=\int_{FT}^{\infty }\omega \left(y_{i}\right)\int_{PT_{i}-y_{i}}^{\infty }\varphi \left(n_{i}\right)dn_{i}dy_{i}dy_{i+1}$$

## 4. The Shannon Entropy of Imperfect Condition Monitoring

As already noted, the events $\Gamma_{1}\left(t_{i},t_{i+1}\right)$, $\Gamma_{4}\left(t_{i},t_{i+1}\right)$, and $\Gamma_{6}\left(t_{i},t_{i+1}\right)$ correspond to the correct decisions, and the events $\Gamma_{2}\left(t_{i},t_{i+1}\right)$, $\Gamma_{3}\left(t_{i},t_{i+1}\right),$ and $\Gamma_{5}\left(t_{i},t_{i+1}\right)$ correspond to incorrect decisions when monitoring the condition of the system at time $t_{i}\;\left(i=1,\;2,\ldots \right)$. With perfect monitoring, the sum of the probabilities of correct decisions would be equal to one, and the sum of the probabilities of incorrect decisions would be zero. Thus, with perfect monitoring, there is no uncertainty. However, real condition monitoring is imperfect due to the non-ideal measuring equipment and various noises. Therefore, the sum of the probabilities of correct decisions is less than unity, and the sum of the probabilities of incorrect decisions is greater than zero. Therefore, any decision made when monitoring the system condition carries some uncertainty. To characterize correct and incorrect decisions, we introduce two indicators, namely, the total error-free and the total error probabilities.

(28) $$P_{error-free}\left(t_{i},t_{i+1}\right)=P\left\{\Gamma_{1}\left(t_{i},t_{i+1}\right)\right\}+P\left\{\Gamma_{4}\left(t_{i},t_{i+1}\right)\right\}+P\left\{\Gamma_{6}\left(t_{i},t_{i+1}\right)\right\}$$

(29) $$P_{error}\left(t_{i},t_{i+1}\right)=P\left\{\Gamma_{2}\left(t_{i},t_{i+1}\right)\right\}+P\left\{\Gamma_{3}\left(t_{i},t_{i+1}\right)\right\}+P\left\{\Gamma_{5}\left(t_{i},t_{i+1}\right)\right\}$$

Further, the degree of uncertainty of the decisions made when monitoring the system condition at the time $t_{i}$ we estimate using Shannon entropy. Substituting $P_{error-free}$ and $P_{error}$ into Equation (1) gives:

(30) $$H\left(t_{i},t_{i+1}\right)=-P_{error-free}\left(t_{i},t_{i+1}\right)\log_{2}P_{error-free}\left(t_{i},t_{i+1}\right)-P_{error}\left(t_{i},t_{i+1}\right)\log_{2}P_{error}\left(t_{i},t_{i+1}\right)$$

Comparing Equations (1) and (30), we can see that m=2, $p_{1}=P_{error-free}$, and $p_{2}=P_{error}$. Moreover, since the events $\bar{\Gamma_{1},\Gamma_{6}}$ form a complete group of incompatible events, then $P_{error-free}+P_{error}=1$. Therefore, due to the properties of Shannon entropy, Equation (30) has a maximum of one bit when $P_{error-free}=P_{error}=0.5$ and tends to zero when $P_{error-free}\to 1$ and $P_{error}\to 0$.

Indicator (30) is a measure of how much information is not available about the system condition.

## 5. Optimal Preventive Maintenance Thresholds

The problem of determining the optimal preventive maintenance threshold $PT_{i}^{opt}$ at inspection time $t_{i}\;\left(i=1,\;2,\ldots \right)$ depends on the chosen optimization criterion. As a criterion for optimizing $PT_{i}^{opt}$ at inspection time $t_{i}$, we choose the minimum of Shannon entropy, i.e.,

(31) $$PT_{i}^{opt}\Rightarrow \underset{PT_{i}}{\min}\left[H\left(t_{i},t_{i+1};PT_{i}\right)\right],\;i=1,\;2,\;\ldots$$

The probabilities $P_{error-free}$ and $P_{error}$ are largely dependent on the preventive maintenance threshold $PT_{i}$ at each time point of condition monitoring. Therefore, choosing the value of the threshold $PT_{i}$ that reduces the probability of $P_{error}$ and increases the probability of $P_{error-free}$, one can achieve a minimum of entropy, i.e., to reach minimum uncertainty at each scheduled time of condition monitoring. This is the meaning of the optimization criterion (31).

## 6. Degradation Process Model

Let us assume that the following monotone stochastic function describes the process of degradation of a system:

(32) $$Y\left(t\right)=a_{0}+A_{1}t^{\beta }$$

where $a_{0}$ is the initial value of the system state parameter *Y*(*t*) defined in the range from 0 to *FT*, $A_{1}$ is the random degradation rate of the system state parameter defined in the interval from 0 to ∞, and β is the exponent of time.

Realizations of the random process of degradation *Y*(*t*) are a convex function, if β > 1, a concave function if β < 1, and a linear function if β = 1.

On the base of the change of variables method [41], we derive the PDF $\omega \left(y_{i}\right)$ and $\omega \left(y_{i},y_{i+1}\right)$ as follows:

(33) $$\omega \left(y_{i}\right)=\frac{1}{t_{i}^{\beta }}\Phi \left(\frac{y_{i}-a_{0}}{t_{i}^{\beta }}\right)$$

(34) $$\omega \left(y_{i},y_{i+1}\right)=\frac{1}{t_{i}^{\beta }}\Phi \left(\frac{y_{i}-a_{0}}{t_{i}^{\beta }}\right)\delta \left\{y_{i+1}-\left[a_{0}+\frac{\left(y_{i}-a_{0}\right)t_{i+1}^{\beta }}{t_{i}^{\beta }}\right]\right\}$$

where Φ($a_{1}$) is the PDF of random degradation rate $A_{1}$ and δ(*x*) is the delta function.

By substitution of Equations (33) and (34) in (17) and (23)–(27), after specific mathematical transformations, we obtain the following analytical formulas for calculating the probabilities of possible decisions when monitoring the system at time $t_{i}$:

(35) $$P\left\{\Gamma_{1}\left(t_{i},t_{i+1}\right)\right\}=\int_{0}^{\left(FT-a_{0}\right)/t_{i+1}^{\beta }}\Phi \left(x\right)\int_{-\infty }^{PT_{i}-(a_{0}+xt_{i}^{\beta })}\varphi \left(n_{i}\right)dn_{i}dx$$

(36) $$P\left\{\Gamma_{2}\left(t_{i},t_{i+1}\right)\right\}=\int_{0}^{\left(FT-a_{0}\right)/t_{i+1}^{\beta }}\Phi \left(x\right)\int_{PT_{i}-(a_{0}+xt_{i}^{\beta })}^{\infty }\varphi \left(n_{i}\right)dn_{i}dx$$

(37) $$P\left\{\Gamma_{3}\left(t_{i},t_{i+1}\right)\right\}=\int_{\left(FT-a_{0}\right)/t_{i+1}^{\beta }}^{\left(FT-a_{0}\right)/t_{i}^{\beta }}\Phi \left(x\right)\int_{-\infty }^{PT_{i}-(a_{0}+xt_{i}^{\beta })}\varphi \left(n_{i}\right)dn_{i}dx$$

(38) $$P\left\{\Gamma_{4}\left(t_{i},t_{i+1}\right)\right\}=\int_{\left(FT-a_{0}\right)/t_{i+1}^{\beta }}^{\left(FT-a_{0}\right)/t_{i}^{\beta }}\Phi \left(x\right)\int_{PT_{i}-(a_{0}+xt_{i}^{\beta })}^{\infty }\varphi \left(n_{i}\right)dn_{i}dx$$

(39) $$P\left\{\Gamma_{5}\left(t_{i},t_{i+1}\right)\right\}=\int_{\left(FT-a_{0}\right)/t_{i}^{\beta }}^{\infty }\Phi \left(x\right)\int_{-\infty }^{PT_{i}-(a_{0}+xt_{i}^{\beta })}\varphi \left(n_{i}\right)dn_{i}dx$$

(40) $$P\left\{\Gamma_{6}\left(t_{i},t_{i+1}\right)\right\}=\int_{\left(FT-a_{0}\right)/t_{i}^{\beta }}^{\infty }\Phi \left(x\right)\int_{PT_{i}-(a_{0}+xt_{i}^{\beta })}^{\infty }\varphi \left(n_{i}\right)dn_{i}dx$$

From the analysis of Equations (35)–(40) follows that the sum of probabilities of correct and incorrect decisions when monitoring the system condition at time $t_{i}$ is equal to unity.

## 7. Results and Discussion

The transmitter is the most expensive part of a radar system [42]. It is of great importance to providing fault prediction; therefore, condition monitoring of the power supply voltage is carried out at discrete times $t_{i}=i\tau \;\left(i=1,\;2,\;\ldots \right)$, where τ is the periodicity of condition monitoring. If the output voltage of the radar transmitter power supply exceeds the threshold *FT* = 25 kV, it enters the failed state, and corrective maintenance is required [42]. The transmitter supply voltage as a function of time is well approximated by the stochastic deterioration process (32) with the following parameter values: $a_{0}=19.645\;\mathrm{kV}$, β=1.3, $E(A_{1})=0.015$ kV/h, $\sigma (A_{1})=0.008$ kV/h, where $E(A_{1})$ and $\sigma (A_{1})$ are, respectively, the mathematical expectation and standard deviation of the random degradation rate $A_{1}$. We further assume that Φ($a_{1}$) is a truncated Gaussian PDF. Accuracy of voltage measurements in the range of 20–30 kV is approximately ±2% [43]. Therefore, we assume that the standard deviation of measurement error $\sigma_{N}=0.4\;\mathrm{kV}$.

**Figure 1:**
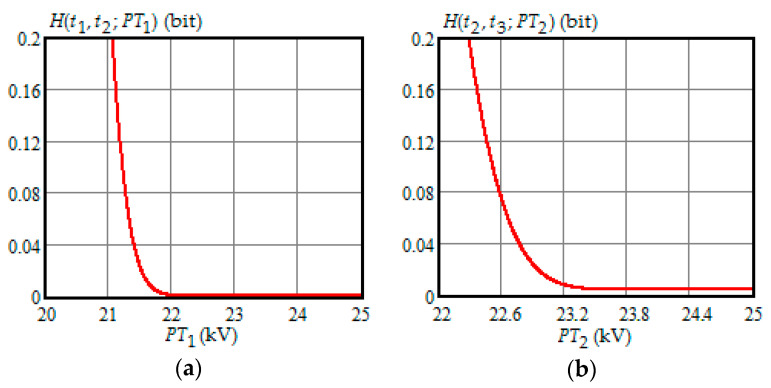
(**a**) The dependence of the Shannon entropy versus preventive maintenance threshold $PT_{1}$ when $t_{1}=100\;h$ and $t_{2}=200\;h$; (**b**) the dependence of the Shannon entropy versus preventive maintenance threshold $PT_{2}$ when $t_{2}=200\;h$ and $t_{3}=300\;h$.

**Figure 2:**
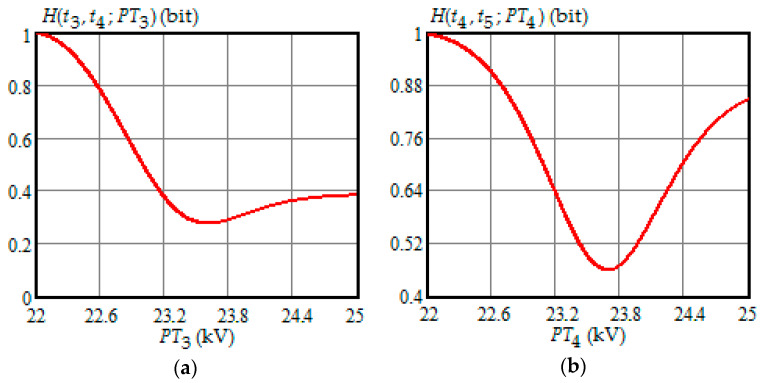
(**a**) The dependence of the Shannon entropy versus preventive maintenance threshold $PT_{3}$ when $t_{3}=300\;h$ and $t_{4}=400\;h$; (**b**) the dependence of the Shannon entropy versus preventive maintenance threshold $PT_{4}$ when $t_{4}=400\;h$ and $t_{5}=500\;h$.

**Figure 3:**
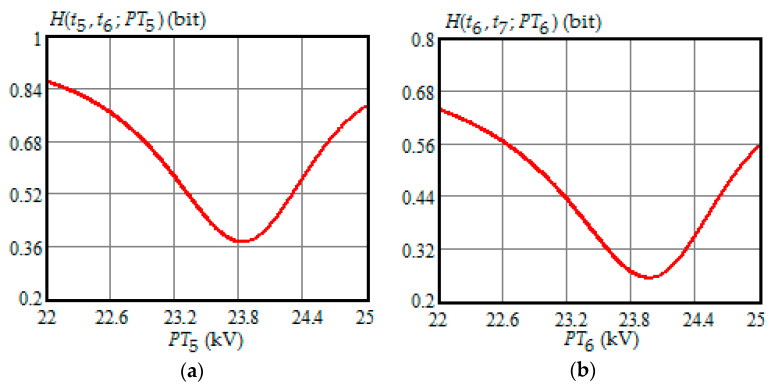
(**a**) The dependence of the Shannon entropy versus preventive maintenance threshold $PT_{5}$ when $t_{5}=500\;h$ and $t_{6}=600\;h$; (**b**) the dependence of the Shannon entropy versus preventive maintenance threshold $PT_{6}$ when $t_{6}=600\;h$ and $t_{7}=700\;h$.

**Figure 4:**
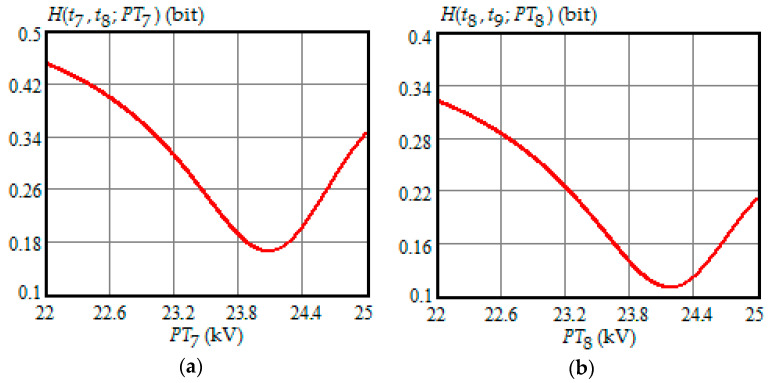
(**a**) The dependence of the Shannon entropy versus preventive maintenance threshold $PT_{7}$ when $t_{7}=700\;h$ and $t_{8}=800\;h$; (**b**) the dependence of the Shannon entropy versus preventive maintenance threshold $PT_{8}$ when $t_{8}=800\;h$ and $t_{9}=900\;h$.

**Figure 5:**
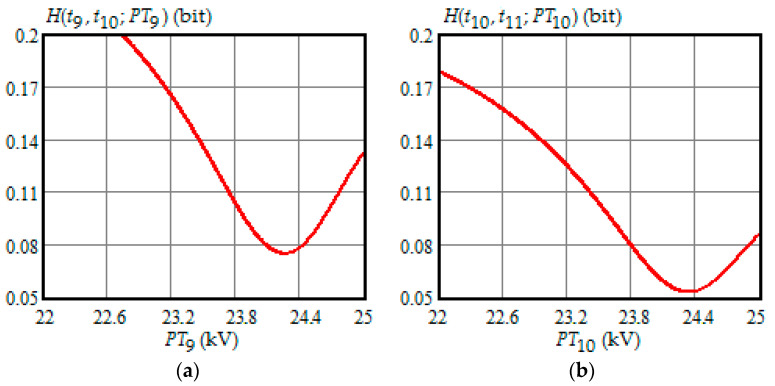
(**a**) The dependence of the Shannon entropy versus preventive maintenance threshold $PT_{9}$ when $t_{9}=900\;h$ and $t_{10}=1000\;h$; (**b**) the dependence of the Shannon entropy versus preventive maintenance threshold $PT_{10}$ when $t_{10}=1000\;h$ and $t_{11}=1100\;h$.

Figure 1, Figure 2, Figure 3, Figure 4 and Figure 5 show the dependence of Shannon entropy on a preventive maintenance threshold for various moments of condition monitoring when τ=100 h. Table 1 shows a summary of the optimization by criterion (31). From Figure 1, Figure 2, Figure 3, Figure 4 and Figure 5 and Table 1, we can draw the following conclusions:

- For moments of condition monitoring $t_{1}$ and $t_{2}$, Shannon entropy decreases with an increase in the preventive threshold and then remains constant up to the failure threshold *FT*. Therefore, as follows from Figure 1a,b, for the moment $t_{1}$ the value of the preventive threshold can be any in the interval (21.9, 25) kV and for the moment $t_{2}$ in the interval (23.3, 25) kV;
- Shannon entropy is a strictly convex function of the preventive maintenance threshold, starting at time $t_{3}\;=\;300\;h$ and subsequent moments of condition monitoring;
- The optimal preventive maintenance threshold increases with the time of inspection for $t_{i}>t_{2}\;\left(i=3,4,\;\ldots \right)$, which may be explained by an increase in the mathematical expectation of the stochastic degradation process (32) with time;
- Starting from time $t_{1}=100\;h$, Shannon’s minimum entropy increases with increasing inspection time, reaching a maximum at $t_{4}=400\;h$, and then decreases almost to zero at $t_{10}=1000\;h$.

**Figure 6:**
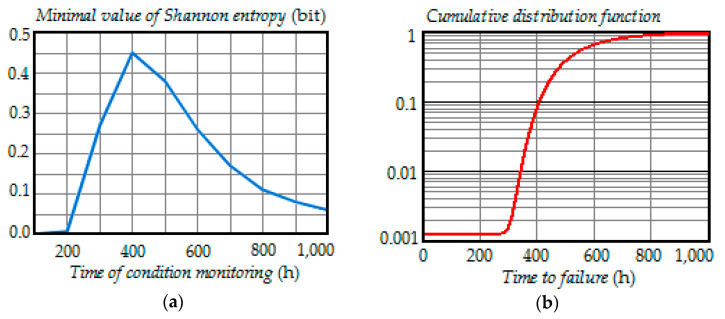
(**a**) The dependence of the minimal Shannon entropy versus time of condition monitoring; (**b**) the dependence of the cumulative distribution function versus time to failure.

To clarify the last conclusion, let us consider simultaneously the dependence of the minimum Shannon entropy on the moment of condition monitoring and the plot of the cumulative distribution function of time to failure, shown in Figure 6a,b. As can be seen in Figure 6a,b, the entropy was nearly zero when the cumulative distribution function was close to zero or unity, respectively, at early and late inspection times.

Indeed, at early inspection times ($t<t_{3}=300\;h$), the item was most probably in the operable state. Therefore, the degree of uncertainty in the condition of the item was low. That is why the Shannon entropy was also low. On the other hand, at late inspection times ($t>t_{7}=700\;h$), the item was most probably in the failed state. Consequently, the degree of uncertainty in the condition of the item was also low. That was why the Shannon entropy was low. Thus, the maximum value of the Shannon entropy corresponded to the time where the cumulative distribution function had the highest rise, i.e., in the vicinity of $t_{4}=400\;h$.

**Figure 7:**
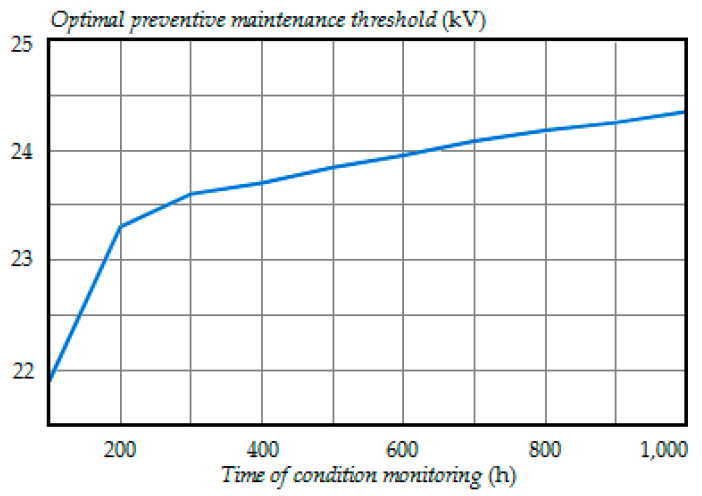
The dependence of the optimal preventive maintenance threshold on the time of condition monitoring.

Figure 7 shows the dependence of the optimal preventive maintenance threshold on the time of condition monitoring. The optimal thresholds for inspection times $t_{1}=100\;h$ and $t_{2}=200\;h$ corresponded to the minimal possible values according to Table 1.

As can be seen in Figure 7, the optimal preventive maintenance threshold increased with the time of condition monitoring gradually approaching the degradation failure threshold *FT*.

The optimal preventive maintenance threshold depends on the time of condition monitoring because of the probabilities of correct and incorrect decisions (35)–(40) that change over time.

**Figure 8:**
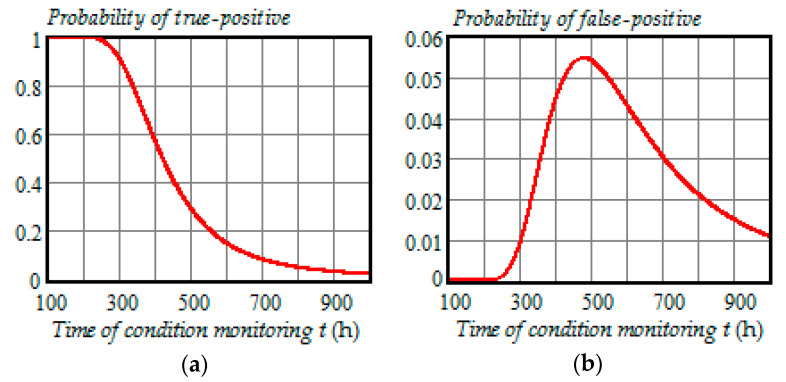
(**a**) Dependence of the probability of true-positive on the time of condition monitoring *t*; (**b**) dependence of the probability of false-positive on the time of condition monitoring *t*.

**Figure 9:**
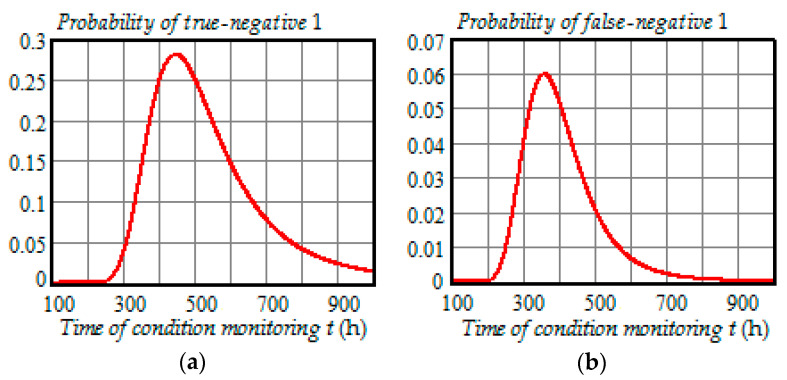
(**a**) Dependence of the probability of true-negative 1 on the time of condition monitoring *t*; (**b**) dependence of the probability of false-negative 1 on the time of condition monitoring *t*.

**Figure 10:**
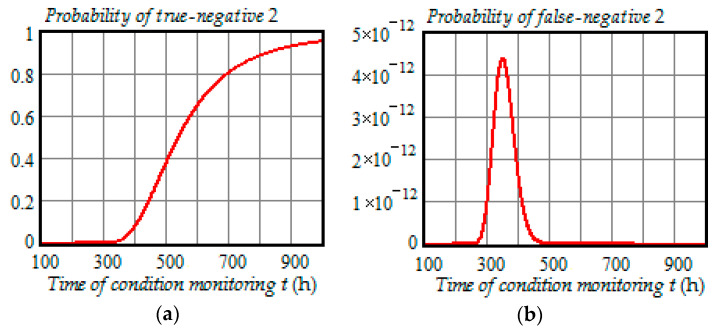
(**a**) Dependence of the probability of true-negative 2 on the time of condition monitoring *t*; (**b**) dependence of the probability of false-negative 2 on the time of condition monitoring *t*.

Figure 8, Figure 9 and Figure 10 illustrate how the probabilities of true-positive, false-positive, true-negative 1, false-negative 1, true-negative 2, and false-negative 2 depend on the time of condition monitoring $t_{i}=t\in \;\left(100\;h,1000\;h\right)$ when the preventive maintenance threshold is 23.7 kV for each time of inspection and $t_{i+1}=t+100\;h$.

From the analysis of plots in Figure 8, Figure 9 and Figure 10, we can draw the following conclusions:

- All probabilities depend on the time of condition monitoring *t*;
- The probability of true-positive is almost constant from 0 to 250 h and starts to decrease rapidly in the interval 300 to 500 h reaching 30% at t=500 h, and then begins to decrease slowly reaching 2.3% at t=1000 h;
- The probability of false-positive begins to go up remarkably at t=240 h and get to 5.5% at t=480 h, and then slowly decreases to 1.1% at t=1000 h;
- The probability of true-negative 1 begins to increase significantly at t=250 h and get to 28% at t=450 h, and then gradually decreases to 1.4% at t=1000 h;
- The probability of false-negative 1 begins to go up strongly at t=100 h and get to 6% at t=360 h, and then decreases to 0.016% at t=1000 h;
- The probability of true-negative 2 is almost zero from 0 to 350 h and starts to increase rapidly in the interval 400 to 600 h reaching 65% at t=600 h, and then increases slower reaching 95.1% at t=1000 h;
- With the chosen preventive maintenance threshold, the probability of false-negative 2 is almost zero over the interval (0, 1000 h).

From Figure 8, Figure 9 and Figure 10, it follows that all the probabilities of correct and incorrect decisions are very much functions of time. Besides, we can see from the PDF (33)–(34) and Formulas (35)–(40) that these probabilities also depend on the model parameters of the degradation process. Therefore, in the CBM models, it is wrong to assume that the probabilities of false-positive, true-positive, false-negative, and true-negative can be constants.

We should note that the proposed approach to decision making at condition monitoring can be applied not only to deteriorating processes described by the model (32) but also to many other monotonic stochastic processes such as the Gamma process, inverse Gaussian process, etc. Specific examples of such processes are the propagation of cracks in the blades of wind turbines [44,45], an increase in the iron content in lubricating oil [46,47], the capacity of lithium-ion batteries [48,49], etc.

## 8. Conclusions

This article proposed a new approach to optimizing the decision-making process when monitoring the condition of a deteriorating system at scheduled times by the criterion of minimum Shannon entropy. Mathematical expressions were derived for evaluating the probabilities of correct and incorrect decisions, such as true-positive, false-positive, true-negative 1, false-negative 1, true-negative 2, and false-negative 2, when monitoring the system condition at a scheduled time. For the first time, the probabilities of correct and incorrect decisions when monitoring the system condition were incorporated into the equation of Shannon entropy. It was first shown that Shannon’s entropy is a convex function of the preventive maintenance threshold for condition monitoring moments. It was also shown that minimal Shannon’s entropy varies from zero at low failure probability to the maximum value at a high rise of failure probability and again drops to almost zero when the cumulative distribution function of time to failure approaches unity. By numerical calculations, it was shown that the optimal preventive maintenance threshold increases with the time of condition monitoring gradually approaching to the degradation failure threshold. For the first time, we showed that the probabilities of true-positive and true-negative 2 are monotonic decreasing and increasing functions of time, respectively; while the probabilities of false-positive, true-negative 1, false-negative 1, and false-negative 2 are not monotonic functions of time. Moreover, the latter four functions have a non-symmetric bell shape with a pronounced maximum. The obtained results can significantly reduce the uncertainty when making decisions about the system condition based on the conducted monitoring.

Our future work will include an application of the proposed approach to different deteriorating systems such as wind turbine blades, gearboxes, and other components; modification of the proposed mathematical model for the case of a multicomponent system; and development of a decision-making model based on imperfect condition monitoring and prognostication.

## Figures and Tables

**Table 1:** Summary of the optimized preventive maintenance thresholds.

Number of Condition Monitoring, *i*	Current Moment of Condition Monitoring, $t_{i}$ (h)	Next Moment of Condition Monitoring, $t_{i+1}$ (h)	Optimal Preventive Maintenance Threshold, $PT_{i}^{opt}$ (kV)	Minimal Value of Shannon Entropy, $H(t_{i},t_{i+1};PT_{i}^{opt})$, (bit)
1	100	200	$21.9<PT_{1}^{opt}\leq 25$	0
2	200	300	$23.3<PT_{2}^{opt}\leq 25$	0.006
3	300	400	23.6	0.27
4	400	500	23.7	0.45
5	500	600	23.84	0.38
6	600	700	23.95	0.26
7	700	800	24.08	0.17
8	800	900	24.18	0.11
9	900	1000	24.25	0.08
10	1000	1100	24.4	0.06

## Abbreviations

The following abbreviations exists in the manuscript:

CBM	Condition-based maintenance
PDF	Probability density function
RUL	Remaining useful life

## Nomenclature

$t_{i}$	Time of conducting condition monitoring
$Y(t_{i})$	Random value of the system state parameter at time $t_{i}$
$\Xi (t_{i})$	Random measurement result of the system state parameter at time $t_{i}$
$N(t_{i})$	Random noise or measurement error at time $t_{i}$
$\xi (t_{i})$	Realization of $\Xi (t_{i})$ at time $t_{i}$
*FT*	Functional failure threshold
$PT_{i}$	Preventive maintenance threshold at time $t_{i}$
$PT_{i}^{opt}$	Optimal preventive maintenance threshold at time $t_{i}$
$\Gamma_{1}\left(t_{i},t_{i+1}\right)$	True-positive event at inspection time $t_{i}$
$\Gamma_{2}\left(t_{i},t_{i+1}\right)$	False-positive event at inspection time $t_{i}$
$\Gamma_{3}\left(t_{i},t_{i+1}\right)$	False-negative 1 event at inspection time $t_{i}$
$\Gamma_{4}\left(t_{i},t_{i+1}\right)$	True-negative 1 event at inspection time $t_{i}$
$\Gamma_{5}\left(t_{i},t_{i+1}\right)$	False-negative 2 event at inspection time $t_{i}$
$\Gamma_{6}\left(t_{i},t_{i+1}\right)$	True-negative 2 event at inspection time $t_{i}$
$P\{\Gamma_{1}\left(t_{i},t_{i+1}\right)\}$	Probability of true-positive event at inspection time $t_{i}$
$P\{\Gamma_{2}\left(t_{i},t_{i+1}\right)\}$	Probability of false-positive event at inspection time $t_{i}$
$P\{\Gamma_{3}\left(t_{i},t_{i+1}\right)\}$	Probability of false-negative 1 event at inspection time $t_{i}$
$P\{\Gamma_{4}\left(t_{i},t_{i+1}\right)\}$	Probability of true-negative 1 event at inspection time $t_{i}$
$P\{\Gamma_{5}\left(t_{i},t_{i+1}\right)\}$	Probability of false-negative 2 event at inspection time $t_{i}$
$P\{\Gamma_{6}\left(t_{i},t_{i+1}\right)\}$	Probability of true-negative 2 event at inspection time $t_{i}$
$\omega (y_{i+1})$	Probability density function of the system state parameter at time $t_{i+1}$
$P_{error-free}\left(t_{i},t_{i+1}\right)$	Total error-free probability
$P_{error}\left(t_{i},t_{i+1}\right)$	Total error probability
*H*($t_{i},t_{i+1}$)	Shannon entropy when monitoring the system condition at the time $t_{i}$
$a_{0}$	Initial value of the system state parameter
$A_{1}$	Random degradation rate of the system state parameter
β	Exponent of time
Φ($a_{1}$)	Probability density function of random degradation rate $A_{1}$
δ(*x*)	Delta function
$E(A_{1})$	Mathematical expectation of the random degradation rate $A_{1}$
$\sigma (A_{1})$	Standard deviation of the random degradation rate $A_{1}$
$\sigma_{N}$	Standard deviation of measurement error

## References

[1] Shannon C. (1948). A mathematical theory of communication. Bell Syst. Tech. J..

[2] Lee K., Lee S.-Y., Kangbin Y. (2019). Machine learning based file entropy analysis for ransomware detection in backup systems. IEEE Access.

[3] Einicke G., Sabti H., Thiel D., Fernandez M. (2018). Maximum-entropy-rate selection of features for classifying changes in knee and ankle dynamics during running. IEEE J. Biomed. Health Inform..

[4] Zu T., Kang R., Wen M., Zhang Q. (2017). Belief reliability distribution based on maximum entropy principle. IEEE Access.

[5] Li H., Pan D., Philip Chen C. (2014). Intelligent prognostics for battery health monitoring using the mean entropy and relevance vector machine. IEEE Trans. Syst. Man Cybern. Syst..

[6] McDonald G., Zhao Q. (2017). Multipoint optimal minimum entropy deconvolution and convolution fix: Application to vibration fault detection. Mech. Syst. Signal Process..

[7] Liu J., Hu Y., Wu B., Jin C. (2017). A hybrid health condition monitoring method in milling operations. Int. J. Adv. Manuf. Technol..

[8] Robles B., Avila M., Duculty F., Vrignat P., Begot S., Kratz F. (2013). Evaluation of minimal data size by using entropy, in a HMM maintenance manufacturing use. IFAC Proc. Vol..

[9] Yankov M., Olsen M., Stegmann M., Christensen S., Forchhammer S. (2019). Fingerprint entropy and identification capacity estimation based on pixel-level generative modelling. IEEE Trans. Inf. Forensics Secur..

[10] Nowak W., Guthke A. (2016). Entropy-based experimental design for optimal model discrimination in the geosciences. Entropy.

[11] Young C., Subbarayan G. (2019). Maximum entropy models for fatigue damage in metals with application to low-cycle fatigue of Aluminum 2024-T351. Entropy.

[12] Liu L., Wang S., Liu D., Zhang Y., Peng Y. (2015). Entropy-based sensor selection for condition monitoring and prognostics of aircraft engine. Microelectron. Reliab..

[13] Liu L., Wang S., Liu D., Peng Y. Quantitative description of sensor data monotonic trend for system degradation condition monitoring. Proceedings of the Prognostics and System Health Management Conference (PHM-Chengdu).

[14] Liu G., Zhao J., Li H., Zhang X. (2019). Bearing degradation assessment based on entropy with time parameter and fuzzy c-means clustering. J. Vibroengineering.

[15] Wang H., Liu Z., Nunez A., Dollevoet R. (2019). Entropy-based local irregularity detection for high-speed railway catenaries with frequent inspections. IEEE Trans. Instrum. Meas..

[16] Aeronautical Design Standard Handbook. Condition-Based Maintenance System for US Army Aircraft: ADS-79D-HDBK. http://everyspec.com/ARMY/ADS-Aero-Design-Std/ADS-79-HDBK_2013_49364/.

[17] Chen N., Ye Z.S., Xiang Y., Zhang L. (2015). Condition-based maintenance using the inverse Gaussian degradation model. Eur. J. Oper. Res..

[18] Abdel-Hameed M. (1987). Inspection and maintenance policies of devices subject to deterioration. Adv. Appl. Probab..

[19] Abdel-Hameed M. (1995). Correction to: “Inspection and maintenance policies of devices subject to deterioration”. Adv. Appl. Probab..

[20] Grall A., Berenguer C., Dieulle L. (2002). A condition-based maintenance policy for stochastically deteriorating systems. Reliab. Eng. Syst. Saf..

[21] Dieulle L., Berenguer C., Grall A., Roussignol M. (2003). Sequential condition-based maintenance scheduling for a deteriorating system. Eur. J. Oper. Res..

[22] Deloux E., Castanier B., Bérenguer C., Kallen M.J., Kuniewski S.P. (2009). An adaptive condition-based maintenance policy with environmental factors. Risk and Decision Analysis in Maintenance Optimization and Flood Management.

[23] Grall A., Dieulle L., Berenguer C., Roussignol M. (2002). Continuous-time predictive-maintenance scheduling for a deteriorating system. IEEE Trans. Reliab..

[24] Huynh K.T., Barros A., Bérenguer C., Castro I. (2011). A periodic inspection and replacement policy for systems subject to competing failure modes due to degradation and traumatic events. Reliab. Eng. Syst. Saf..

[25] Wang H.K., Huang H.Z., Li Y.F., Yang Y.J. (2016). Condition-based maintenance with scheduling threshold and maintenance threshold. IEEE Trans. Reliab..

[26] Guo C., Bai Y., Jia Y. Maintenance optimization for systems with non-stationary degradation and random shocks. Proceedings of the 9th IMA International Conference on Modelling in Industrial Maintenance and Reliability.

[27] Liu B., Xie M., Kuo W. Condition-based maintenance for degrading systems with state-dependent operating cost. Proceedings of the 9th IMA International Conference on Modelling in Industrial Maintenance and Reliability.

[28] Flage R., Coit D.W., Luxhoj J.T., Aven T. (2012). Safety constraints applied to an adaptive Bayesian condition-based maintenance optimization model. Reliab. Eng. Syst. Saf..

[29] Deloux E., Castanier B., Bérenguer C. (2012). Environmental information adaptive condition-based maintenance policies. Struct. Infrastruct. Eng..

[30] He K., Maillart L.M., Prokopyev O.A. (2015). Scheduling preventive maintenance as a function of an imperfect inspection interval. IEEE Trans. Reliab..

[31] Kallen M., Noortwijk J. (2005). Optimal maintenance decisions under imperfect inspection. Reliab. Eng. Syst. Saf..

[32] Newby M., Dagg R. Optimal inspection policies in the presence of covariates. Proceedings of the European Safety and Reliability Conference (ESREL’02).

[33] Ye Z., Chen N., Tsui K.L. (2015). A Bayesian approach to condition monitoring with imperfect inspections. Qual. Reliab. Eng. Int..

[34] Tang S., Yu C., Wang X., Guo X., Si X. (2014). Remaining useful life prediction of lithium-ion batteries based on the Wiener process with measurement error. Energies.

[35] Lam Y. (2003). An inspection-repair-replacement model for a deteriorating system with unobservable state. J. Appl. Probab..

[36] Badıa F., Berrade M.D., Campos C.A. (2002). Optimal inspection and preventive maintenance of units with revealed and unrevealed failures. Reliab. Eng. Syst. Saf..

[37] Berrade M., Cavalcante A., Scarf P. (2012). Maintenance scheduling of a protection system subject to imperfect inspection and replacement. Eur. J. Oper. Res..

[38] Zequeira R.I., Berenguer C. (2006). Optimal scheduling of non-perfect inspections. IMA J. Manag. Math..

[39] Berrade M.D., Scarf P.A., Cavalcante C.A.V., Dwight R.A. (2013). Imperfect inspection and replacement of a system with a defective state. A cost and reliability analysis. Reliab. Eng. Syst. Saf..

[40] Alaswad S., Xiang Y. (2017). A review on condition-based maintenance optimization models for stochastically deteriorating system. Reliab. Eng. Syst. Saf..

[41] Walpole R., Myers R., Myers S., Ye K. (2012). Probability and Statistics for Engineers and Scientists.

[42] Ma C., Shao Y., Ma R. (2013). Analysis of equipment fault prediction based on metabolism combined model. J. Mach. Manuf. Autom..

[43] 80K-40 High Voltage Probe-Fluke Corporation. https://dam-assets.fluke.com/s3fs-public/80k40___iseng0900.pdf.

[44] Besnard F., Bertling L. (2010). An approach for condition-based maintenance optimization applied to wind turbine blades. IEEE Trans. Sustain. Energy.

[45] Sutherland H. (2000). A Summary of the fatigue properties of wind turbine materials. Wind Energy.

[46] Wang Z., Xue X., Yin H., Jiang Z., Li Y. (2018). Research progress on monitoring and separating suspension particles for lubricating oil. Complexity.

[47] Coronado D., Fisher K. Condition Monitoring of Wind Turbines: State of the Art, User Experience, and Recommendations. Project Report. https://www.semanticscholar.org/paper/CONDITION-MONITORING-OF-WIND-TURBINES-%3A-STATE-OF-%2C-CoronadoFischer/477fabdc00482a7f1265efc5fbc5ee15db66d353.

[48] Sood B., Severn L., Osterman M., Pecht M., Bougaev A., McElfresh D. Lithium-ion battery degradation mechanisms and failure analysis methodology. Proceedings of the 38th International Symposium for Testing and Failure Analysis.

[49] Zhai Q., Ye Z.-S. (2017). RUL prediction of deteriorating products using an adaptive Wiener process model. IEEE Trans. Ind. Inform..

